# Multiple Lacunar Cerebral Infarcts as the Initial Presentation of COVID-19

**DOI:** 10.7759/cureus.9638

**Published:** 2020-08-10

**Authors:** Amro Elshereye, Burak Erdinc

**Affiliations:** 1 Internal Medicine, Brookdale University Hospital and Medical Center, Brooklyn, USA

**Keywords:** covid-19, sars-cov-2, acute ischemic stroke, lacunar infarct

## Abstract

Coronavirus Disease-19 (COVID-19) is a novel corona virus that started as an outbreak in the Hubei province of China in December 2019 and later became a pandemic affecting every continent on the planet. Patients with severe COVID-19 tend to develop acute thrombotic complications including myocardial infarction, pulmonary embolism, and ischemic stroke. We describe a case of a 75-year-old-female who presented with acute onset slurred speech and right sided facial droop. She was diagnosed with COVID-19 with acute ischemic stroke as the initial presentation. Stroke as the initial presentation of COVID-19 is rare and has not been described in the literature frequently. The purpose of this report is to raise awareness about this potential complication of COVID-19 as an initial presentation.

## Introduction

As of July 9, 2020 there exists 11,874,226 million cases of coronavirus disease-19 (COVID-19) with a mortality of 545,481 thousand cases worldwide [1]. COVID-19 is a novel coronavirus that started as an outbreak in the Hubei province of China in December 2019 and later became a pandemic affecting every continent on the planet. The earliest spread was related to zoonotic or environmental exposure associated with a seafood market event in Wuhan, but it quickly became evident that human to human spread is occurring [2]. The most common symptoms of COVID 19 are fever, cough, fatigue, and myalgias [3]. COVID-19 tends to have a more severe clinical course in patients who are elderly or with comorbidities including hypertension, diabetes mellitus and coronary heart disease [3]. Patients with severe COVID-19 tend to develop acute thrombotic complications including myocardial infarction, pulmonary embolism and ischemic stroke [4]. It has been shown that COVID-19 can present with neurological symptoms such as cerebrovascular accident (CVA), ataxia, headaches, anosmia and impaired consciousness [5]. Stoke is now a recognizable complication of severe COVID 19 and we need more data to help with treatment and prevention of this COVID 19 complication [6]. We here represent a rare case of COVID-19 presenting as an acute embolic stroke.

## Case presentation

A 75-year-old female with only a known past medical history of hypertension only was brought in by emergency medical services (EMS) as a prenotification for stroke. She was found locked inside her apartment unable to answer the door for her home health aid. She was found to have acute onset slurred speech and right-sided facial droop and was brought to Brookdale University Hospital and Medical Center emergency department. Patient's initial vital signs showed a temperature of 101.8 °F, blood pressure of 145/71 mm Hg, heart rate of 71 beats per minute and respiratory rate of 20 breath per minute, oxygen saturation of 85% on room air. Neurological exam revealed, slurred speech, right facial weakness, muscle strength of 3/5 on both upper extremities and 2/5 on both lower extremities, right upper extremity pronation drift. She was able to follow simple commands, had intact sensation, no gaze deviation or dysmetria. Remainder of her physical exam did not reveal any other abnormal findings. Patient’s National Institutes of Health Stroke scale was 13 (scores range from 0 to 42, with higher numbers indicating greater stroke severity).

**Figure 1:**
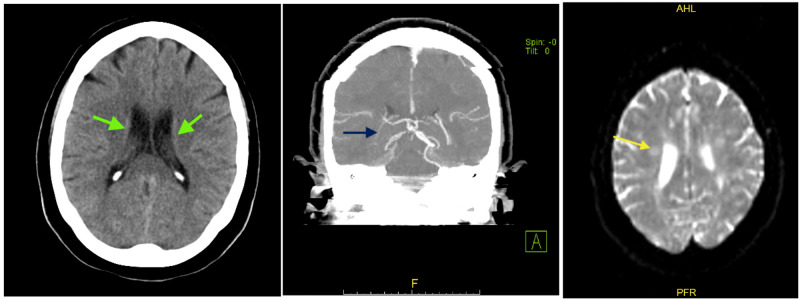
Computed tomography (CT) of the head without contrast showing mild periventricular white matter disease (left, green arrows). CT angiogram of the head and neck with contrast showed non-opacification of the right vertebral artery suggesting occlusion (middle, blue arrow). Magnetic resonance imaging (MRI) of the brain showing multiple small areas of restricted diffusion within both frontal and parietal lobes the largest located within the right frontal lobe measuring 9 mm (right, yellow arrow)

CT of the head without contrast showed mild periventricular white matter disease with no other abnormal findings (Figure 1). CT angiogram of the head and neck with contrast showed non-opacification of the right vertebral artery suggesting occlusion, patent carotid arteries and left vertebral artery (Figure 1). Patient was not a candidate for tissue plasminogen activator since her last known well before arrival to the hospital was unknown. She was evaluated by neuro interventional team and was not found to be a candidate for catheter directed thrombectomy. MRI of the brain was done four hours later and it showed multiple small areas of restricted diffusion within both frontal and parietal lobes the largest located within the right frontal lobe measuring 9 mm. These are likely due to multiple focal areas of acute lacunar infarctions (Figure 1).

**Figure 2:**
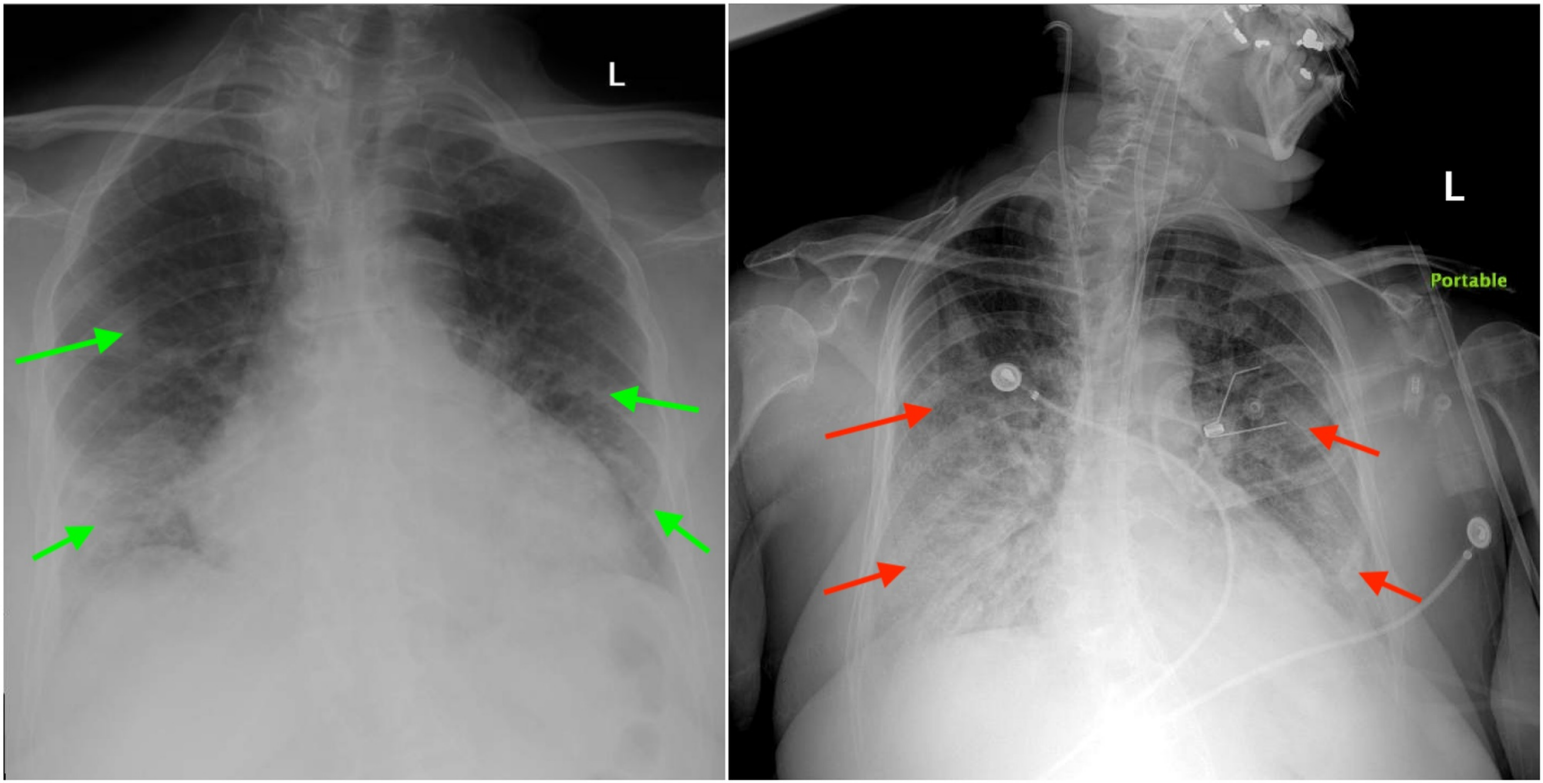
Initial chest x-ray showing bilateral airspace disease (left, green arrows). Post intubation chest x-ray on day three showing endotracheal tube and increased bilateral infiltrates in comparison to the chest x-ray on admission (right, red arrows)

Initial pertinent laboratory investigations revealed acute kidney injury and high inflammatory markers as summarized in Table 1. Complete blood panel and the rest of the chemistry were within normal limits. A chest X-ray showed bilateral airspace disease (Figure 2). Severe acute respiratory syndrome coronavirus 2 (SARS-COV-2) nasopharyngeal swab was sent.

**Table 1:** Initial laboratory investigations revealing acute kidney injury and high inflammatory markers

Chemistry	Result	Reference values
Blood urea nitrogen	30.0 mg/dL	9 – 20 mg/dL
Creatinine	1.73 mg/dL	0.66 – 1.25 mg/dL
Lactate dehydrogenase	1095 IU/L	313 – 618 IU/L
C-reactive protein	22.0 mg/dL	0.50 – 1.00 mg/dL
Ferritin	650.0 ng/mL	11.10 – 264.00 ng/mL
D-dimer	468 ng/mL	0 – 230 ng/mL

The patient was admitted to the neuroscience unit for further management. She was started on aspirin, high-intensity statin for acute ischemic stroke management and hydroxychloroquine 400 mg twice a day for one day followed by 200 mg twice a day for four days, azithromycin 500 mg tablets daily and ceftriaxone 1 g intravenous daily for the empiric treatment community-acquired pneumonia and suspected SARS-COV2 infection. On day two of admission, her oxygen requirement increased, and the patient started using 10 liters of oxygen via non-rebreather mask. Her SARS-COV-2 PCR test resulted positive and ceftriaxone and azithromycin therapy, which was started empirically for bacterial pneumonia, was discontinued on day two. Her speech markedly improved on day three. The patient was able to pass the bedside swallow test and started having a pureed diet with aspiration precautions. Her acute kidney injury resolved as well with intravenous hydration. On third night of her admission, the patient’s oxygen saturation dropped to 56% despite being on 15 liters oxygen treatment via non-rebreather mask and the patient had to be intubated for hypoxic respiratory failure. Patient was intubated by anesthesiology and started on mechanical ventilation. Her post-intubation chest x-ray showed endotracheal tube in the proper position and increased bilateral infiltrates in comparison to the chest x-ray on admission (Figure 2). Patient was transferred to the medical intensive care unit for further management of hypoxic respiratory failure secondary to SARS-CoV-2 pneumonia. She finished a five-day course of hydroxychloroquine and azithromycin treatment. On day six, her course was complicated by acute kidney failure which was thought to be pre-renal in etiology. On day eight, the patient had a cardiac arrest with initial rhythm revealing asystole. Cardiopulmonary resuscitation was started immediately without return of spontaneous circulation (ROSC) and the patient expired.

## Discussion

Many theories have been suggested as to why COVID 19 patients can get CVA as a complication. One of the proposed mechanisms involves Angiotensin-converting enzyme 2 (ACE-2). ACE-2 was found to be the functional receptor for SARS-COV 2 virus [7]. ACE-2 is expressed in multiple organ systems including the lung alveoli and the endothelium of blood vessels. It is thought that COVID 19 binding to ACE-2 causes depletion of ACE-2 and thus the counterregulatory mechanism of ACE-2 on the renin angiotensin system (RAS) leading to a procoagulant environment predisposing to venous and arterial thrombosis [8]. In addition, it is suggested that cytokine storm caused by severe SARS-CoV-2 infection can precipitate acute thrombotic events. It becomes evident by the elevated inflammatory markers which cause a pro-inflammatory medium that predisposes patients to thrombosis and in turn neurological complication such as acute stroke [9].

The incidence of stroke in hospitalized patients with COVID 19 is 1.6% overall and 3.7% in patients admitted to the intensive care unit (ICU) [6]. Acute CVA appears to occur more frequently in patients with severe COVID 19 as compared to patients with nonsevere COVID 19 [5]. This alerts us to the fact that patients who present with stroke as an initial presentation for COVID 19 like our patient should be monitored in a more intensive setting as it is very likely that they will have a more severe clinical course.

The management of stroke in patients with COVID 19 should still follow the same guidelines as patients without COVID 19 with adequate infection control measures [10]. TPA and catheter directed thrombolysis remain the treatment of choice for patients with ischemic CVA in COVID 19 patients [11]. The concern lies more in the rapid response due to lack of PPE or having adequate diagnostic modalities that can preserve respiratory isolation [12]. We hope with multiple reports about CVA of stroke in COVID 19 patients that clinicians will be more aware of this clinical entity.

## Conclusions

Acute stroke is one of many neurological complications that can arise in a patient who is afflicted with COVID 19. We believe that clinicians should be alert to the fact that COVID 19 might present with CVA or other neurological manifestations as to not delay diagnosis and appropriate isolation of those patients. CVA tends to happen more often in patients with severe COVID 19 as opposed to nonsevere COVID 19. We believe that recognizing this complication is important since it can result in rapid clinical deterioration in those patients.
